# Network interactions with functional roles and evolutionary relationships for BURP domain-containing proteins in chickpea and model species

**DOI:** 10.6026/973206300191197

**Published:** 2023-12-31

**Authors:** Khela Ram Soren, Sandhya Tripathi, Maloti Hembram, Neeraj Kumar, Aravind Konda K, NC Gupta, Chellapilla Bharadwaj, Girish Prasad Dixit

**Affiliations:** 1 ICAR-Indian Institute of Pulses Research, Kanpur, India; 2 ICAR-Division of genetics, IARI, New Delhi, India; 3 University of Delhi, New Delhi, India; 4 National Institute of Plant Biotechnology, New Delhi, India

**Keywords:** Network interaction, BURPs, STRING database, *Cicer arietinum*, salinity stress

## Abstract

The functional significance and evolutionary relationships of BURP domain-containing genes unique to plants is of interest. Network analysis reveals different associations of BURP proteins with other proteins and functional terms, throwing light on their involvement in various biological processes and pathways. The gene expression data reveals that BURP genes are affected by salinity stress, reflecting diverse expression patterns in roots and shoots.

## Background:

BURP domain-containing genes, unique to plants, are intricately involved in the processes of plant development and stress responses [1]. The BURP domain-containing protein, encoded by a stress-responsive gene family, is exclusive to plants and plays significant roles in both plant development and stress response [2, 3]. The BURP domain-containing proteins share specific conserved modules comprising of an N-terminal hydrophobic domain with a potential transit peptide, a short conserved or other segment, an optional segment consisting of unique repeated units for each BURP-domain-containing family member, and finally, the C-terminal BURP domain [4, 5]. The BURP domain is approximately 230 amino acids long and typically includes highly conserved sequences, such as two phenylalanine (F) residues, two cysteine (C) residues, and four repeated cysteine-histidine (CH) motifs [4]. In general, the sequence conforms to CHXCHX23-27CHX23-26CHX8W, where X denotes any amino acid residue [6]. Multiple experimental studies provide substantial evidence supporting the presence of BURP domain responsible for multidimensional actions performed by its representative members, indicating that these proteins operate in a synchronized and collaborative manner [7,8, 9].This domain is named after four typical members - BNM2, USP, RD22, and PG1β and is a conserved C-terminal protein domain exclusively present in plants, indicating towards its plant-specific functions [10, 11]. Here, B represents BNM2 which is specifically expressed during microspore embryogenesis in oilseed rape [12]. U is for USPs expressed during early stages of zygotic and *in vitro* embryogenesis in field bean, R denotes RD22 which acts as a dehydration-responsive protein in **Arabidopsis* thaliana*, while P signifies PG1β, a non-catalytic β-subunit of poly-galacturonase isozyme 1, expressed during tomato (*Solanum lycopersicum*) ripening [13,14].

Salinity corresponds to a significant abiotic stress responsible for significant agricultural losses globally [15]. Among the affected crops, chickpea (*Cicer arietinum* L.) claims great importance as a beneficial legume crop [16- 17,18,19]. However, chickpea is observed as particularly sensitive to salt stress [20]. The adverse effects of salinity on chickpea growth and yield present significant challenges for its cultivation in regions exposed to high soil salinity [21,22]. Addressing the salt sensitivity of chickpea and designing strategies to uplift its tolerance to salinity are essential steps towards sustaining chickpea production and safeguarding food supplies in salt-affected agricultural areas [23-24, 25,26].

Biological processes depend on intricate networks of proteins and their interactions. These protein-protein interactions (PPI) are structured as PPI networks [27,28]. To collect PPI data, both wet-lab and computational techniques are used, and specialized databases like DIP, STRING, and BioGRID retain this information [29,30]. Among these databases, STRING is strikingly important due to its extensive coverage, data abundance, and stringent quality control of PPI data [31, 32]. It compiles PPIs from various sources, including experimental and computational methods, and assigns a combined quality score for each interaction, incorporating data from literature and gene expression profiles [33].

Within PPI networks, modules exist, representing distinct collections of proteins with specific functions or phenotypes [34]. Analysing these modules allows for the identification of underlying protein interactions that dictate molecular functions and phenotypic outcomes [35]. Additionally, PPI networks are invaluable for predicting novel protein candidates associated with certain functions or phenotypes, based on their interactions with known neighbouring proteins [36,37]. While wet-lab methods are available for such predictions, computational methods offer advantages in terms of speed, cost-effectiveness, and reduced labour compared to wet-lab approaches [38,39]. Therefore, it is of interest to decode network interactions with functional roles and evolutionary relationships for BURP domain-containing proteins in chickpea and model species.

## Materials and Methods:

## Data mining and network plotting:

We obtained completely assembled and annotated BURP proteins of *Cicer arietinum* from Phytozome database [40]. The STRING database (Version 9.0) was utilized to acquire interaction data for individual BURP proteins [41]. The search specifically focused on experimentally validated protein-protein interactions for BURP proteins. STRING incorporates information from various sources, including the Protein Data Bank (PDB), Biological General Repository for Interacting Datasets (BIOGRID), Molecular Interaction Database - European Bioinformatics Institute (IntAct-EBI), Molecular Interaction Database (MINT), Biomolecular Interaction Network Database (BIND), European Molecular Biology Laboratory (EMBL) and the Database of Interacting Proteins (DIP) [42-43,44, 45,47, 48]. To organize and extract information about the proteins identified in the BURP network, Gene ontology database, INTERPRO for domain prediction and the Kyoto Encyclopedia of Genes (KEGG) for pathway mapping, were utilized [49,50,51].

## Gene expression data:

Initially, we downloaded the raw data from the Gene Expression Omnibus database. We also performed Gene Ontology term and Kyoto Encyclopedia of Genes and Genomes pathway enrichment analyses to gain insights into the biological functions and pathways associated with the DEGs. The expression data thus obtained was used to generate heat maps using TBTools v.1.120 [52, 53].

## Synteny and collinearity analysis:

Analysis of synteny relationships for BURP proteins between various plant species was observed to find the species closely related to *Cicer arietinum*. Higher stringency parameters were applied to reflect proximity with chickpea BURP proteins. Data was plotted for proteins with similarity upto 75 percent, 99 percent and 50 percent. Collinearity diagram was plotted for model plant species using MCScanX tool and Dual synteny plotter features available in TBtoolsv1.120.

## Results:

To represent different associations with their respective metrics, including the number of nodes, number of edges, average node degree, average local clustering coefficient, expected number of edges, and PPI (Protein-Protein Interaction) enrichment p-value the table was drawn. It displayed various associations labelled from 1 to 7, along with their corresponding network metrics. Each association represents a biological network with a fixed number of nodes and edges. The average node degree, which measures the average number of edges connected to each node, ranges from 1.5 to 6.19. The average local clustering coefficient (indicating the density of connections between a node's neighbours) ranges from 0.75 to 0.905, suggesting varying degrees of clustering in the networks. The expected number of edges is also given for each association, estimating the number of edges based on the network's characteristics. Additionally, the PPI enrichment p-value is provided, denoting the statistical significance of the associations in terms of protein-protein interaction enrichment. The data in the table represents important insights into the structure and connectivity of biological networks, shedding light on their functional relationships and potential significance in biological processes (Table 1). Further, major gene ontology descriptions and associated KEGG pathway details for various BURP associations were represented collectively in a table. The table includes data on gene annotations related to Gene Ontology (GO) components, GO functions, GO processes, and KEGG pathways. For GO components, "Ribosome" (GO:0005840) shows 5 observed genes out of 413 background genes with a strength of 1.43 and a low false discovery rate of 6.30E-04. GO functions "Glutamate 5-kinase activity" (GO: 0004349) and "glutamate-5-semialdehyde dehydrogenase activity" (GO: 0004350) both exhibit 3 observed genes out of 6 background genes, with strengths of 3.05 and false discovery rates of 1.27E-05. Another GO function, "Structural constituent of ribosome" (GO:0003735), displays 5 observed genes out of 368 background genes, with a strength of 1.48 and a false discovery rate of 7.60E-04. Furthermore, various GO processes are listed, such as "L-proline biosynthetic process" (GO: 0055129) with 3 observed genes out of 8 background genes and a false discovery rate of 6.84E-05. Additionally, KEGG pathways like "Arginine and proline metabolism" (cam00330) and "Biosynthesis of amino acids" (cam01230) exhibit 3 observed genes out of 55 and 214 background genes, respectively, with varying strengths and false discovery rates (Table 2).

## Clusters associations within BURP domain proteins:

The clustering coefficient is a significant network parameter used to quantify the tendency of nodes/proteins in a graph to form clusters or groups. This measure is crucial as it offers valuable insights into the overall organization of relationships within the network. Moreover, it can indicate the existence of functional modules. In protein networks, these modules may represent higher-order complexes or signalling pathways, revealing the intricate interactions and potential functional associations among proteins. Following are the major associations occurring within BURP domain protein members in *C. arietinum.*

## Association 1:

**Figure 1:**
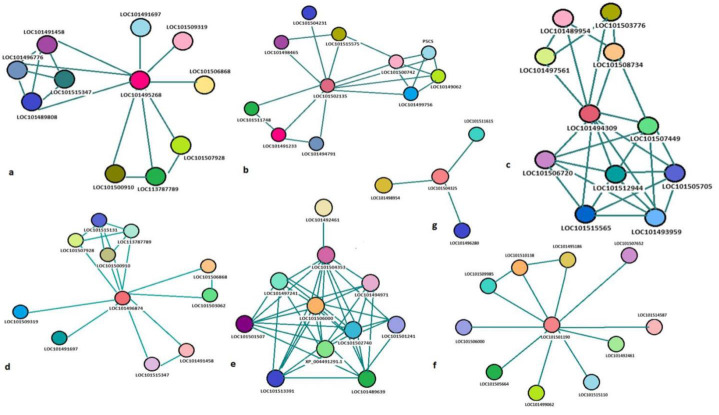
Clusters Associations within BURP domain Proteins (a.) association of two USP1-l & two BNM2A-like BURP domain containing protein with seven other proteins.(b.) association of RD22, VF3.01&USP-like BURP domain containing protein with other biosynthesis proteins.(c.) association of BURP domain 9-like protein with regulatory& biosynthesis proteins.(d.) association of BNM2A-like BURP domain protein with AMS proteins.(e.) association of Polygalactouronase-1 non-catalytic subunit beta-like BURP domain protein.(f.) association of Polygalactouronase-1 non-catalytic subunit beta-like BURP domain protein with regulatory genes.(g.) association of embryonic abundant protein VF30.1-like BURP domain protein with carotenoid biosynthesis genes.

The four BURPs reported as XP_004507419.1 (BURP domain protein USPL1-like/BURP domain containing protein 3-like), XP_12567688.1 (BURP domain-containing protein BNM2A-like), XP_004500818.1 (BURP domain protein USPL1-like), XP_012567687.1 (BURP domain-containing protein BNM2A-like) were shown to be linked with the network related to comprising items such as mixed, incl. arbuscular mycorrhizal association, BURP domain, and AT-hook motif nuclear-localized protein 15-29, transcription factor that specifically binds AT-rich DNA sequences related to the nuclear matrix attachment regions (MARs), and lipopolysaccharide binding. The major proteins observed in this association were named as putative bpi/lbp family protein At1g04970-like, uncharacterized protein loc101491458; upstream in-frame stop codon, probable poly-galacturonase at1g80170-like isoform X1; belongs to the glycosyl hydrolase 28 family, serine carboxypeptidase-like clade ii; serine carboxypeptidase-like 40-like; belongs to the peptidase s10 family, protein dcl, chloroplastic-like; upstream in-frame stop codon and uncharacterized protein LOC101491803 (Figure 1a).

## Association 2:

This association contains four BURP proteins XP_004498543.1 (BURP domain protein RD22), XP_0045111751.1 (embryonic abundant protein VF3.01-like), XP_004487743.1 (unknown seed protein USP-like) plotted to be linked with a network comprising terms like L-proline biosynthetic process, transpiration, glutamate 5-kinase activity, glutamate-5-semialdehyde dehydrogenase activity, Mixed, incl. transpiration, and BURP domain, proline biosynthesis, arginine and proline metabolism, biosynthesis of amino acids, proline biosynthesis, ATP-binding, amino acid kinase family, aldehyde dehydrogenase family, gpr domain, glutamate/acetylglutamate kinase, glutamate 5-kinase/delta-1-pyrroline-5-carboxylate synthase, delta l-pyrroline-5-carboxylate synthetase, glutamate 5-kinase, conserved site, gamma-glutamyl phosphate reductase gpr, conserved site, aspartate/glutamate/uridylate kinase, acetylglutamate kinase-like superfamily, aldehyde dehydrogenase domain, aldehyde dehydrogenase, n-terminal, aldehyde dehydrogenase, C-terminal, and aldehyde/histidinol dehydrogenase. Proteins involved were delta-1-pyrroline-5-carboxylate synthase-like, ACT domain-containing protein ACR12; unknown protein DS12 from 2D-PAGE of leaf, chloroplastic-like, ABC transporter G family member 22-like, Protein RSI-1-like, low-temperature-induced 65 kDa protein-like, dehydration-responsive protein RD22-like, uncharacterized protein LOC101504231; protein DR_1172-like, ABC transporter G family member 22-like isoform X1, chaperone protein ClpD, chloroplastic-like; belongs to the ClpA/ClpB family(Figure 1b).

## Association 3:

Association 3 comprised of the BURP protein XP_004507416.1 (BURP domain-containing protein 9) linked with the terms such as regulation of cellular process, regulation of transcription, dna-templated, heterochromatin maintenance, regulation of circadian rhythm chromo/chromo shadow domain, and SUVR5, C2H2-type Zinc Figure 3 repeats, SWI/SNF complex, and BRIGHT, ARID (A/T-rich interaction domain) domain, Cullin, N-terminal, and Ubiquitin-conjugating enzyme, active site, HMG (high mobility group) box, HMG-box domain, dimerisation domain, Skp1 family, tetramerization domain, POZ domain, S-phase kinase-associated protein 1-like, , chromodomain-helicase-dna-binding protein 3-like, chromatin structure-remodeling complex protein syd-like isoform x1, protein phosphatase 2c and cyclic nucleotide-binding/kinase domain-containing protein; upstream in-frame stop codon, high mobility group b protein 15-like isoform x1, high mobility group b protein 10-like isoform X1(Figure 1c).

## Association 4:

Here BURP protein XP_004500819.1 (BURP domain-containing protein BNM2A-like) was associated with the terms such as mixed, incl. arbuscular mycorrhizal association, and BURP domain, AT-hook motif nuclear-localized protein. The major proteins involved in the network were uncharacterized protein LOC101491458; upstream in-frame stop codon, Probable poly-galacturonase At1g80170-like isoform X1; Belongs to the glycosyl hydrolase 28 family, Dehydration-responsive protein RD22-like; BURP domain-containing protein 17-like, Putative DNA-binding protein ESCAROLA-like, Probable WRKY transcription factor 53-like, Spermidine synthase-like, uncharacterized protein loc101509319; Coilin; Upstream in-frame stop codon, Uncharacterized protein LOC101515131, Protein dcl, chloroplastic-like; upstream in-frame stop codon, uncharacterized protein LOC101491803 (Figure 1d).

## Association 5:

This association has shown the presence of BURP related protein XP_004514685.1 (Polygalactouronase-1 non-catalytic subunit beta-like), XP_004514684.1 (Polygalactouronase-1 non-catalytic subunit beta-like), XP_004509826.1 (Polygalactouronase-1 non- beta-like protein 3), XP_004509821.1 (Polygalactouronase-1 non- beta-like protein 3) containing the gene ontology terms such as translation, chloroplast accumulation movement, chloroplast avoidance movement, structural constituent of ribosome, ribosome, mixed, incl. WEB family, and BURP domain, hexitol dehydrogenase activity, and BURP domain, ribosomal protein, ribosomal protein L16p/L10e, weak chloroplast movement under blue light. The proteins present in the network were weak chloroplast movement under blue light 1-like, tetraketide alpha-pyronereductase 1-like, polygalacturonase-1 non-catalytic subunit beta-like isoform X1, 54S ribosomal protein L39, mitochondrial-like; 50S ribosomal protein L33-like etc. (Figure 1e).

## Association 6:

The BURP protein XP_004501939.1 Polygalactouronase-1 non- beta-like protein 3 here was related to the terms mixed, incl. hydroquinone glucosyltransferase activity, and atpase-coupled xenobiotic transmembrane transporter activity, auxin canalisation, plant pleckstrin homology-like region, domain of unknown function DUF828 and plant VAN3-binding protein. Proteins observed in the network were tetraketide alpha-pyronereductase 1-like, protein NRT1/ PTR FAMILY 6.4, nitrate transporter 1.3-like, uncharacterized protein LOC101499062 (Figure 1f).

## Association 7:

BURP protein XP_004490752.1 (embryonic abundant protein VF30.1-like), XP_004490674.1 (embryonic abundant protein VF30.1-like) lastly, was linked to the terms carotene catabolic process, carotenoid dioxygenase activity, low-temperature-induced 78kDa/65kDa, and BURP domain, carotenoid biosynthesis, plastoglobule dioxygenase, retinal pigment epithelial membrane protein, carotenoid oxygenase. Major protein players in this group were probable carotenoid cleavage dioxygenase 4, chloroplastic-like, probable glycosyl-transferase at5g03795-like; belongs to the glycosyltransferase 47 family, embryonic abundant protein VF30.1-like (Figure 1g).

## Clustered gene expression under salinity stress:

The expression patterns of the candidate genes observed in cluster association analysis under salinity stress was retrieved from Gene Expression Omnibus database (GEO). The retrieved data expression was accordingly represented using the heat maps and is described in the following expression datasets.

## Gene expression dataset 1:

**Figure 2:**
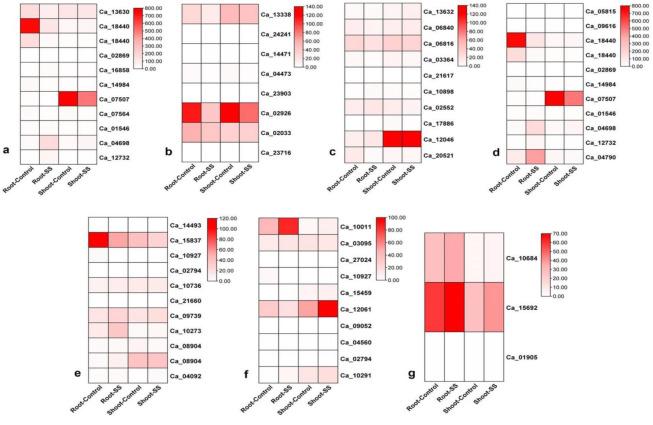
Expression profiles of chickpea BURP genes in various tissues under salinity. The FPKM 48 values were displayed for gene expression levels based on the publicly available transcriptome data to depict the heat map.

Among the genes analyzed, Ca_13630 showed relatively higher expression in the root under control conditions (158.859) compared to the expression level in the root under salinity stress (80.0822). Similarly, the shoot of *Cicer arietinum* exhibited higher expression of Ca_13630 under control conditions (113.85) compared to the expression under salinity stress (114.994). Gene Ca_18440 displayed significantly higher expression in the root under control conditions (738.892) as opposed to the expression in the root under salinity stress (117.174). In the shoot, Ca_18440 showed higher expression under control conditions (34.0369) than under salinity stress (46.439). Additionally, there is another entry for Ca_18440, suggesting multiple observations for this gene with different expression values. Another gene, Ca_02869, exhibited low expression in both root and shoot under control conditions (0.683797 and 0.259986, respectively). Notably, its expression level was zero in both root and shoot under salinity stress. For Ca_16858, higher expression was observed in the root under salinity stress (14.9134) compared to control (8.53602). However, in the shoot, the expression was higher under control conditions (4.78837) than under salinity stress (5.19128). Gene Ca_14984 showed higher expression in the root under control conditions (22.1619) compared to its expression in the root under salinity stress (9.72882). In the shoot, the expression of Ca_14984 was higher under control conditions (10.8599) than under salinity stress (8.53696). On the other hand, Ca_07507 exhibited higher expression in the root under control conditions (12.3086) compared to the root under salinity stress (5.32589). However, the shoot displayed significantly higher expression of Ca_07507 under salinity stress (757.683) compared to its expression under control conditions (501.505). Gene Ca_07564 exhibited higher expression in the root under control conditions (16.3639) compared to its expression under salinity stress (9.84369). Similarly, in the shoot, the expression of Ca_07564 was higher under control conditions (20.5998) than under salinity stress (10.953). Ca_01546 showed higher expression in the root under control conditions (10.1333) compared to its expression under salinity stress (14.5885). However, in the shoot, the expression was higher under salinity stress (10.4736) than under control conditions (5.39248). Another gene, Ca_04698, displayed higher expression in the root under control conditions (19.197) compared to its expression under salinity stress (170.097). In the shoot, the expression of Ca_04698 was higher under control conditions (25.531) compared to its expression under salinity stress (50.0954). Lastly, gene Ca_12732 exhibited higher expression in the root under control conditions (1.12487) compared to its expression under salinity stress (43.1854). Moreover, the expression of Ca_12732 was almost negligible in both root and shoot under salinity stress (0.165601 and 0.143318, respectively, (Figure 2a).

## Gene expression dataset 2:

Gene Ca_13338 exhibited higher expression in the root under control conditions (32.5185) compared to its expression in the root under salinity stress (16.9837). Similarly, in the shoot, the expression of Ca_13338 was higher under control conditions (57.717) compared to its expression under salinity stress (50.6604). Gene Ca_24241 showed no expression (value of 0) in all samples, indicating that this gene might not be actively transcribed under the given conditions. Gene Ca_14471 displayed no expression in the root under control conditions but showed low expression (0.050073) in the root under salinity stress. In the shoot, Ca_14471 exhibited slightly higher expression under control conditions (0.0508717) compared to its expression under salinity stress (0).Gene Ca_04473 exhibited higher expression in both root and shoot under control conditions (3.09098 and 5.87786, respectively) compared to its expression under salinity stress (1.16719 and 1.72726, respectively). Gene Ca_02926 displayed significantly higher expression in both root and shoot under control conditions (126.073 and 135.631, respectively) compared to its expression under salinity stress (47.2969 and 93.5025, respectively).Gene Ca_02033 exhibited higher expression in both root and shoot under control conditions (65.0445 and 37.3162, respectively) compared to its expression under salinity stress (47.609 and 39.6197, respectively).Gene Ca_23716 showed no expression (value of 0) in all samples, indicating that this gene might not be actively transcribed under the given conditions (Figure 2b).

## Gene expression dataset 3:

Among the genes analysed, Ca_13632 showed relatively higher expression in the root under control conditions (4.6786) compared to the expression level in the root under salinity stress (3.63312). Similarly, the shoot of *Cicer arietinum* exhibited higher expression of Ca_13632 under control conditions (8.32876) compared to the expression under salinity stress (12.027).Gene Ca_06840 displayed higher expression in both root and shoot under control conditions (13.293 and 12.3142, respectively) compared to the expression under salinity stress (7.69974 and 15.5592, respectively).Another gene, Ca_06816, exhibited higher expression in both root and shoot under control conditions (29.9193 and 34.7757, respectively) compared to the expression under salinity stress (22.0839 and 32.2982, respectively).On the other hand, gene Ca_03364 showed higher expression in the shoot under control conditions (8.70013) compared to the expression in the shoot under salinity stress (5.75684). However, the root displayed higher expression of Ca_03364 under control conditions (5.2682) compared to its expression under salinity stress (2.23737).Two genes, Ca_21617 and Ca_17886, showed no expression (expression value of 0) in all samples, indicating that these genes might not be actively transcribed under the given conditions. Gene Ca_10898 displayed higher expression in the root under salinity stress (3.2133) compared to the expression under control conditions (0.997685). However, in the shoot, the expression of Ca_10898 was higher under control conditions (2.89461) compared to its expression under salinity stress (1.95969).

Gene Ca_02552 exhibited higher expression in both root and shoot under control conditions (13.047 and 13.0529, respectively) compared to the expression under salinity stress (17.8701 and 9.53234, respectively).Gene Ca_12046 displayed significantly higher expression in both root and shoot under control conditions (12.3065 and 121.074, respectively) compared to the expression under salinity stress (17.6769 and 121.306, respectively).Finally, gene Ca_20521 showed higher expression in the root under control conditions (15.3176) compared to its expression under salinity stress (4.03426). In the shoot, the expression of Ca_20521 was also higher under control conditions (7.47862) than under salinity stress (6.25404, Figure 2c).

## Gene expression dataset 4:

Gene Ca_09616 showed no expression (expression value of 0) in the root under control conditions but displayed low expression in the root under salinity stress (0.289709). Additionally, in the shoot, Ca_09616 exhibited low expression under control conditions (0.144529) but no expression (value of 0) under salinity stress. As for gene Ca_18440, it showed significantly higher expression in the root under control conditions (738.892) compared to the expression in the root under salinity stress (117.174). Similarly, in the shoot, the expression of Ca_18440 was higher under control conditions (34.0369) compared to its expression under salinity stress (46.439). Additionally, there is another entry for Ca_18440, suggesting multiple observations for this gene with different expression values. Gene Ca_02869 exhibited low expression in both root and shoot under control conditions (0.683797 and 0.259986, respectively). Interestingly, its expression level was zero in both root and shoot under salinity stress. Gene Ca_14984 showed higher expression in the root under control conditions (22.1619) compared to its expression in the root under salinity stress (9.72882). In the shoot, the expression of Ca_14984 was higher under control conditions (10.8599) than under salinity stress (8.53696). Gene Ca_07507 exhibited higher expression in the root under control conditions (12.3086) compared to the root under salinity stress (5.32589). However, in the shoot, the expression of Ca_07507 was significantly higher under salinity stress (757.683) compared to its expression under control conditions (501.505). Gene Ca_01546 showed higher expression in the root under control conditions (10.1333) compared to its expression under salinity stress (14.5885). However, in the shoot, the expression was higher under salinity stress (10.4736) than under control conditions (5.39248). Gene Ca_04698 displayed higher expression in the root under control conditions (19.197) compared to its expression under salinity stress (170.097). In the shoot, the expression of Ca_04698 was higher under control conditions (25.531) compared to its expression under salinity stress (50.0954).

Lastly, gene Ca_12732 exhibited higher expression in the root under control conditions (1.12487) compared to its expression under salinity stress (43.1854). Moreover, the expression of Ca_12732 was almost negligible in both root and shoot under salinity stress (0.165601 and 0.143318, respectively). Additionally, gene Ca_04790 showed significantly higher expression in both root and shoot under control conditions (96.3268 and 38.3284, respectively) compared to the expression under salinity stress (412.351 and 23.2623, respectively, Figure 2d).

## Gene expression dataset 5:

Among the genes analysed, gene Ca_14493 exhibited no expression (expression value of 0) in all samples, indicating that this gene might not be actively transcribed under the given conditions. Gene Ca_15837 showed significantly higher expression in the root under control conditions (102.825) compared to the expression in the root under salinity stress (53.7996). Similarly, in the shoot, the expression of Ca_15837 was higher under control conditions (39.9165) compared to its expression under salinity stress (27.9955). Gene Ca_10927 displayed low expression in the root under control conditions (4.22199) but no expression (value of 0) under salinity stress. Additionally, in the shoot, Ca_10927 showed no expression under both control and salinity stress conditions, except for a minimal expression value of 0.0704156 under salinity stress. Similarly, gene Ca_02794 exhibited no expression (value of 0) in all samples, indicating that this gene might not be actively transcribed under the given conditions. Gene Ca_10736 displayed slightly higher expression in the root under control conditions (8.83521) compared to its expression under salinity stress (11.4296). In the shoot, the expression of Ca_10736 was slightly higher under control conditions (13.8488) compared to its expression under salinity stress (13.2816).

Gene Ca_21660 showed no expression (value of 0) in all samples, indicating that this gene might not be actively transcribed under the given conditions. Gene Ca_09739 exhibited higher expression in both root and shoot under control conditions (16.5644 and 17.0362, respectively) compared to its expression under salinity stress (27.183 and 20.8742, respectively).Gene Ca_10273 showed higher expression in both root and shoot under control conditions (13.762 and 4.26468, respectively) compared to the expression under salinity stress (35.0915 and 4.97925, respectively).Gene Ca_08904 displayed slightly higher expression in the root under control conditions (2.82354) compared to its expression under salinity stress (3.11496). However, in the shoot, the expression was higher under control conditions (7.2004) than under salinity stress (3.41023). Additionally, there is another entry for Ca_08904, suggesting multiple observations for this gene with different expression values. In this second observation, the expression of Ca_08904 was higher in both root and shoot under control conditions (4.05751 and 38.431, respectively) compared to the expression under salinity stress (9.02339 and 37.003, respectively).Lastly, gene Ca_04092 exhibited slightly higher expression in both root and shoot under control conditions (1.98705 and 1.15122, respectively) compared to its expression under salinity stress (3.37766 and 2.627, respectively, Figure 2e).

## Gene expression dataset 6:

Gene Ca_10011 exhibited significantly higher expression in the root under control conditions (42.4711) compared to its expression in the root under salinity stress (85.8192). Similarly, in the shoot, the expression of Ca_10011 was higher under control conditions (4.5579) compared to its expression under salinity stress (9.9853).Gene Ca_03095 showed higher expression in both root and shoot under control conditions (12.567 and 16.7934, respectively) compared to its expression under salinity stress (15.4074 and 14.3992, respectively).Gene Ca_27024 showed no expression (value of 0) in all samples, indicating that this gene might not be actively transcribed under the given conditions. Gene Ca_10927 displayed low expression in the root under control conditions (4.22199) but no expression (value of 0) under salinity stress. Additionally, in the shoot, Ca_10927 showed no expression under both control and salinity stress conditions, except for a minimal expression value of 0.0704156 under salinity stress. Gene Ca_15459 exhibited no expression (value of 0) in the root under control conditions but displayed low expression in the root under salinity stress (0.128077). In the shoot, Ca_15459 showed higher expression under control conditions (6.97976) compared to its expression under salinity stress (8.44777). Gene Ca_12061 displayed higher expression in both root and shoot under control conditions (31.0092 and 51.5441, respectively) compared to its expression under salinity stress (18.7319 and 96.4971, respectively).Gene Ca_09052 showed no expression (value of 0) in all samples, indicating that this gene might not be actively transcribed under the given conditions. Gene Ca_04560 displayed low expression in both root and shoot under control conditions (0.0512002 and 0.591975, respectively) and under salinity stress (0.119736 and 0.520124, respectively).Similarly, gene Ca_02794 exhibited no expression (value of 0) in all samples, indicating that this gene might not be actively transcribed under the given conditions. Lastly, gene Ca_10291 exhibited higher expression in both root and shoot under control conditions (0.630205 and 15.2122, respectively) compared to its expression under salinity stress (5.01513 and 19.3333, respectively, Figure 2f).

Gene Ca_10684 exhibited higher expression in the root under control conditions (27.0441) compared to its expression in the root under salinity stress (35.7971). Similarly, in the shoot, the expression of Ca_10684 was higher under control conditions (3.7272) compared to its expression under salinity stress (4.8495).Gene Ca_15692 showed higher expression in both root and shoot under control conditions (58.2327 and 27.874, respectively) compared to its expression under salinity stress (69.7648 and 39.6699, respectively).Gene Ca_01905 displayed low expression in the root under control conditions (0.375314) and no expression (value of 0) in the root under salinity stress. In the shoot, Ca_01905 showed slightly higher expression under control conditions (0.422354) compared to its expression under salinity stress (0.288445, Figure 2g).

## Synteny analysis:

A map depicting the physical locations of CaBURP genes revealed their distribution across the chromosomes and scaffolds in C. arietinum.

**Figure 3:**
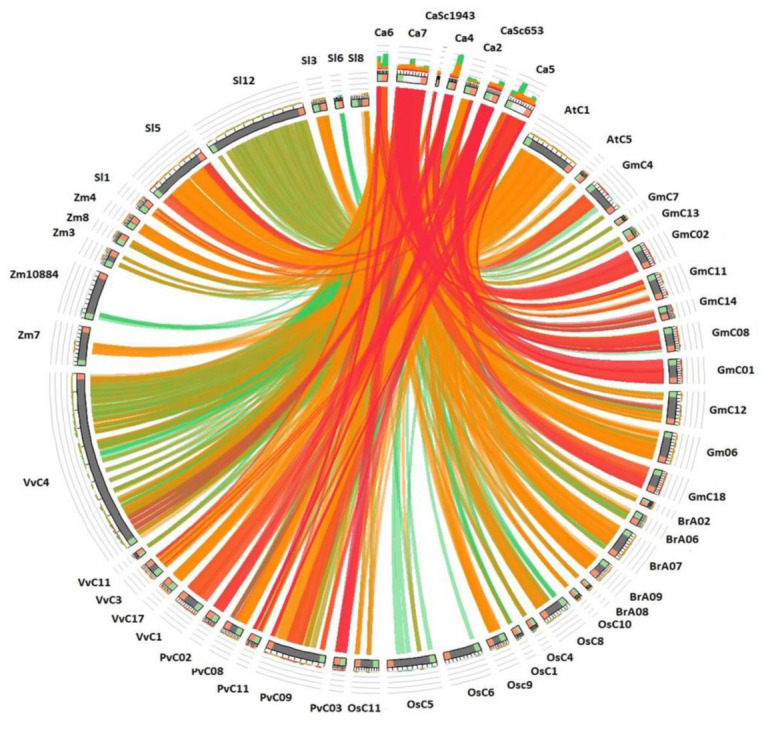
Circos plot showing orthologs between *C. arietinum*, *S. lycopersicum*, *Z. mays*, *V. vinifera*, *P. vulgaris*, *O. sativa*, *B. rapa*, *G. max* and *A. thaliana*.

Each gene was plotted based on its location on specific chromosome and scaffold and reflecting its syntenic association with orthologous genes that matching BURP gene on respective chromosome of other comparative species such as *S. lycopersicum*, *Z. mays*, V. vinifera, *P. vulgaris*, *O. sativa*, *B. rapa*, *G. max* and *A. thaliana* including the species in which BURP genes were identified initially. The plotted species were selected based on the tops hit similarity analysis of BURP genes across various plant species. The distribution of the 15 CaBURP genes was uneven, with varying gene counts on different chromosomes. Chr7 had the highest number of genes followed by Chr5, scaffold653, chr2, chr6, chr4 and scaffold1943 lastly. The maximum region of CaBURP similarity was observed with chromosome 4 of *V. vinifera* followed by chromosome 12 of *S. lycopersicum*, chromosome 5 of *S. lycopersicum* , chromosome 9 of *P. vulgaris* and chromosome 1 of *A. thaliana* respectively. A significant region of similarity was also observed in scaffold 884 of *Z. mays*, chromosome 5 of *O. sativa*, chromosome 7 of *B. rapa*, chromosome 1, 6 and 2 of *G. max* and so on (Figure 3).

## Co-linearity analysis:

To investigate the phylogenetic mechanisms of the BURP family further, a collinearity diagram was constructed, comparing *C. arietinum* with two model species: *A. thaliana* and *M. truncatula*. The analysis revealed that 15 BURP genes showed collinear relationships with genes from the two species: *A. thaliana* (7) and *M. truncatula* (12). Thel BURP genes were associated with syntenic gene pairs, between A. thaliana and *M. truncatula* suggesting that these genes play significant roles in evolution. The gene AT1G23760.1.TAIR10 in *Arabidopsis* is found to be collinear with the gene Ca_07503.v1.0.492 on chromosome 5 in *C. arietinum*, indicating a conserved relationship between these genes. Similarly, genes on chromosome 1 of *Arabidopsis*, namely AT1G70370.1.TAIR10 and AT1G60390.1.TAIR10, are also collinear with the gene Ca_07503.v1.0.492 on chromosome 5 in *C. arietinum*, suggesting the preservation of these gene pairs across the two species. Additionally, the gene AT1G49320.1.TAIR10 on chromosome 1 in *Arabidopsis* exhibits collinearity with the gene Ca_13632.v1.0.492 on chromosome 6 in *C. arietinum*, further highlights the conserved genomic relationships between *Arabidopsis* and *C. arietinum.* Moreover, the gene AT1G23760.1.TAIR10 on chromosome 1 of *Arabidopsis* is collinear with the gene Ca_14493.v1.0.492 on chromosome 7 in *C. arietinum.* Similarly, the genes AT1G70370.1.TAIR10 and AT1G60390.1.TAIR10 on chromosome 1 of *Arabidopsis* also show collinearity with the gene Ca_14493.v1.0.492 on chromosome 7 in *C. arietinum.*

**Figure 4:**
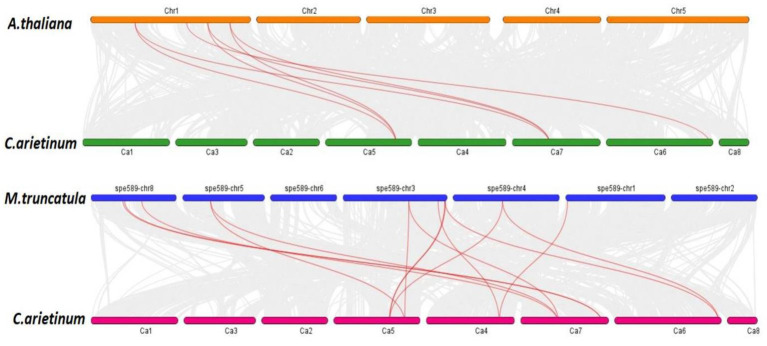
A co-linearity relationship of 15 BURP genes of *C. arietinum* comparing with two model species: *A. thaliana* and *M. truncatula*.

On chromosome 8 of *M. truncatula*, three genes - Medtr8g044290.1.JCVIMt4.0v1, Medtr8g045880.1.JCVIMt4.0v1, and Medtr8g064500.1.JCVIMt4.0v1 - were found to be collinear with the gene Ca_15745.v1.0.492 on chromosome 7 of *C. arietinum.* This collinearity suggests that these specific BURP genes have conserved genomic positions and potential functional relationships between M. truncatula and *C. arietinum.* Similarly, on chromosome 5 of *M. truncatula*, the gene Medtr5g034320.1.JCVIMt4.0v1 was observed to be collinear with two genes in *C. arietinum*: Ca_07503.v1.0.492 on chromosome 5 and Ca_14493.v1.0.492 on chromosome 7. This collinearity indicates shared evolutionary history and conserved genomic regions among these gene pairs across the two plant species. On chromosome 3 of *M. truncatula*, several collinear relationships were observed. The gene Medtr3g109490.1.JCVIMt4.0v1 was found to be collinear with the gene Ca_23903.v1.0.492 on chromosome 4 of *C. arietinum.* Additionally, the gene Medtr3g078090.1.JCVIMt4.0v1 exhibited collinearity with the gene Ca_07503.v1.0.492 on chromosome 5, and the gene Medtr3g116410.1.JCVIMt4.0v1 was collinear with the gene Ca_04789.v1.0.492 on chromosome 5 in *C. arietinum.* Furthermore, the gene Medtr3g116270.1.JCVIMt4.0v1 was found to be collinear with two genes in C. arietinum: Ca_04790.v1.0.492 on chromosome 5 and Ca_13632.v1.0.492 on chromosome 6. Finally, the gene Medtr3g078090.1.JCVIMt4.0v1 was also collinear with the gene Ca_14493.v1.0.492 on chromosome 7 in *C. arietinum.* These collinear relationships highlight the conservation of genomic regions and potential functional significance of these BURP genes between the two species. On chromosome 4 of *M. truncatula*, the gene Medtr4g069520.2.JCVIMt4.0v1 was found to be collinear with two genes in *C. arietinum*: Ca_04790.v1.0.492 on chromosome 5 and Ca_13632.v1.0.492 on chromosome 6, indicating shared evolutionary history between these genes in both species. Lastly, on chromosome 1 of *M. truncatula*, the gene Medtr1g008420.1.JCVIMt4.0v1 was observed to be collinear with the gene Ca_23903.v1.0.492 on chromosome 4 of *C. arietinum*, suggesting conserved genomic regions between *M. truncatula* and *C. arietinum* in this region (Figure 4).

## Discussion:

Crop plants are constantly faced with a variety of environmental stresses, which can significantly impede their growth and, in turn, adversely affect their economic yield. Salt stress poses a major challenge as an abiotic stress factor, significantly impacting global agriculture production [54,55]. Among the affected crops, chickpea stands out as particularly sensitive to salt stress at different growth stages. To address this issue and enhance agricultural resilience, a deeper understanding of salt tolerance in chickpea is essential, as it paves the way for targeted breeding efforts aimed at developing salt-tolerant chickpea varieties [56,57]. By understanding the specific stressors and their effects on crop plants, we can develop targeted approaches to enhance resilience and improve crop yields, ultimately ensuring food security and sustainable agricultural practices [58, 59]. Previous studies on BURP genes have predominantly focused on the RD22 and USP-like subfamily, leaving a limited understanding of the broader BURP gene family in plants [58]. With the advent of new generation sequencing, genome-wide analyses of various genes and transcription factors have increased, but BURP genes have received less attention. Only a few plant species, such as rice, maize, grapevine, soybean, cotton, sorghum, and poplar, have been subjected to extensive and comprehensive genome-wide BURP gene studies [60]. The regulatory function of BURP-like proteins is evolutionarily conserved across lower and higher plants, demonstrating a shared response among various plant classes to diverse environmental challenges. These proteins play a crucial role in coordinating and modulating biological processes to cope with different stressors [61,62]. In one of our previous investigation of the chickpea crop, we have functionally characterized RD22, a BURP gene candidate in *C. arietinum* (Unpublished). Overall, our study contributes to a more comprehensive understanding of the BURP gene family in plants, highlighting the significance of gene duplication events and chromosome-specific expansions in shaping the evolution of BURP genes. To gain a comprehensive understanding of the interconnectedness of BURP and related signalling pathways, a systems-level approach was employed. This approach involved analyzing data on direct and indirect interactions to construct a protein interaction network specifically for BURP expressed under abiotic stress. The data used in this network was carefully curated from the Search Tool for the Retrieval of Interacting Genes (STRING) database, ensuring that only experimentally validated interactions were included. By utilizing the network method, it becomes possible to visualize both the overall structure and the underlying organization of protein-protein interactions related to BURP proteins. The constructed interaction network consists of 7 associations, 70 nodes and 143 edges, exhibiting a scale-free topology. Additionally, this study identified collinear gene pairs among other model species like *A. thaliana* and M. truncatula. The observed networks are associated with various biological terms such as ribosome, glutamate 5-kinase activity, glutamate-5-semialdehyde, dehydrogenase activity, structural constituent of ribosome, carotenoid dioxygenase activity, l-proline biosynthetic process, transpiration, regulation of cellular process, regulation of transcription, DNA-templated, heterochromatin maintenance, regulation of circadian rhythm, translation, chloroplast accumulation movement, chloroplast avoidance movement, carotene catabolic process, arginine and proline metabolism, biosynthesis of amino acids. The study also highlights bottleneck proteins that serve as bridges between signalling pathways and BURP proteins that appear in networks. Furthermore, comprehensive functional annotations are provided for all proteins present in the network. The purpose of the study was to explore the identification of key pathways and hub genes associated with functions of BURP genes by analyzing the expression of associated genes in the network in salinity treated chickpea root and shoot tissues. Through functional enrichment analysis, we discovered a significant association between BURPs and other proteins in the network. These networks are closely associated with the BURP and partner proteins in the interaction pathway and have significant implications for providing leads towards their further functional characterization. Our study successfully covered significant pathways and genes that are strongly associated with BURP proteins. The study of these networks and genes has great potential to advance our understanding of the underlying mechanisms involved in BURP proteins under abiotic stress i.e. salinity. By investigating these networks and exploring the functions of the participating genes, we can gain valuable insights into the abiotic stress mechanisms in chickpea.

Earlier it was deduced that BURP proteins play a conserved role in stress signal transduction across the plant kingdom, achieved through their interactions with MKK proteins in protein-protein interactions [61]. The present study describes different associations of BURP proteins with other proteins and functional terms. These associations were observed within the context of a network analysis, and each association is linked to specific functional terms or biological processes. The first association involves four BURP proteins (XP_004507419.1, XP_12567688.1, XP_004500818.1, XP_012567687.1) and their connections to a network comprising various terms, such as "Mixed," "arbuscular mycorrhizal association," "lipopolysaccharide binding," and "AT-hook motif nuclear-localized proteins." Additionally, several major proteins, including BURP domain proteins and others involved in transcription regulation and enzymatic activities, are present in this network. The second association includes four BURP proteins (XP_004498543.1, XP_0045111751.1, XP_004487743.1, XP_004509826.1) and their connections to terms related to "L-proline biosynthetic process," "Transpiration," and "Arginine and proline metabolism." The network also contains proteins with functions in proline biosynthesis, protein kinases, and dehydration-responsive proteins. Next to this third association involves the BURP protein XP_004507416.1 and its connections to terms related to "regulation of transcription," "heterochromatin maintenance," and "chromatin organization." The network includes proteins associated with transcription factors and chromatin modifiers. The fourth association centres around the BURP protein XP_004500819.1 and its links to terms like "arbuscular mycorrhizal association" and "AT-hook motif nuclear-localized proteins." The network contains proteins associated with transcription factors and chloroplast-related functions. Furthermore, the fifth association includes BURP proteins (XP_004514685.1, XP_004514684.1, XP_004509826.1, XP_004509821.1) and their connections to terms related to "translation," "ribosomal proteins," and "chloroplast movement." The network contains ribosomal proteins and proteins involved in chloroplast movement. Association 6 involves the BURP protein XP_004501939.1 and its links to terms related to "auxin canalization" and "plant pleckstrin homology-like region." The network contains proteins associated with nitrate transporters and chloroplast-related functions. The last association centres around BURP proteins (XP_004490752.1, XP_004490674.1) and their connections to terms related to "carotenoid biosynthesis" and "carotenoid dioxygenase activity." The network includes carotenoid-related proteins and enzymes involved in carotenoid metabolism. Overall, these associations provide insights into the potential functional roles of BURP proteins in diverse biological processes and pathways. The network analysis helps to elucidate the intricate interactions among BURP proteins and other proteins, shedding light on their involvement in various cellular functions and biological processes. Further experimental investigations of these associations may lead to a better understanding of BURP protein functions and their significance in plant development and stress responses.

The plant-specific BURP-containing protein family plays a crucial role in enabling plants to adapt to challenging environmental conditions. Understanding how plants cope with adverse environments heavily relies on information about protein-protein interactions, as it serves as a significant avenue for unravelling these adaptive mechanisms [63,64]. These versatile signalling pathways are integral to the overall adaptability and survival of plants in ever-changing environmental conditions. Present analysis also describes gene expression patterns of BURP proteins under salinity stress in *Cicer arietinum* (chickpea). The gene expression data was retrieved from the Gene Expression Omnibus (GEO) database and represented using heat maps. The expression patterns of several BURP genes were compared between control conditions and salinity stress conditions in both roots and shoots. Here gene expression changes were revealed by the gene expression data which shows that the expression of various BURP genes in the roots and shoots of chickpea plants was influenced by salinity stress. Some genes exhibited higher expression levels under control conditions, while others showed higher expression under salinity stress. Specifically, some genes have shown notable expression pattern. For example, the gene Ca_13630 displayed higher expression in the root and shoot under control conditions compared to salinity stress. In contrast, gene Ca_07507 showed higher expression in the root under control conditions but had significantly higher expression in the shoot under salinity stress. Some genes, like Ca_18440 and Ca_08904, had multiple entries, indicating that they were observed multiple times with different expression values. This suggests possible gene expression variability under the given conditions. Some genes, such as Ca_23903, Ca_21617, Ca_17886, Ca_14493, Ca_21660, Ca_02794, Ca_09052, and Ca_27024, showed no expression in all samples, suggesting that these genes might not be actively transcribed under the conditions tested. The expression patterns of certain genes, like Ca_10898 and Ca_10273, were different in the root and shoot under control conditions and salinity stress. This suggests tissue-specific responses to salinity stress. The data here was divided into multiple gene expression datasets, each representing different sets of genes and experimental conditions. These datasets provide valuable information on the expression patterns of specific BURP genes under salinity stress. Major functional implications of the differential gene expression dataset suggests the changes in gene expression patterns under salinity stress and indicate the involvement of BURP proteins in the plant's response to stress. BURP proteins might play roles in stress adaptation, signal transduction, and other physiological processes related to salinity stress. Overall, the gene expression data provides valuable insights into the differential expression of BURP genes under salinity stress, highlighting the complex regulatory mechanisms that plants employ to cope with environmental challenges. Further experimental studies and functional analyses are necessary to fully understand the roles of BURP proteins in the response to salinity stress in chickpea and other plants.

Synteny analysis describes the results of a study that mapped the physical locations of CaBURP genes across the chromosomes and scaffolds in the species. We also investigated the syntenic association of CaBURP genes with matching BURP genes in other plant species, including *S. lycopersicum* (tomato), *Z. mays* (maize), *V. vinifera* (grapevine), *P. vulgaris* (common bean), *O. sativa* (rice), *B. rapa* (Chinese cabbage), *G. max* (soybean), and *A. thaliana* (thale cress). The distribution of CaBURP genes across chickpea chromosomes and scaffolds was found to be uneven. Some chromosomes and scaffolds contained a higher number of BURP genes compared to others. This uneven distribution may suggest differences in the regulation and functional roles of BURP genes on different chromosomes and scaffolds. Chromosomes with Chromosome 7 (Chr7) in chickpea had the highest number of CaBURP genes, followed by Chromosome 5 (Chr5). This higher gene count on specific chromosomes could indicate the importance of these chromosomes in terms of stress response or other physiological processes where BURP genes are involved. We analyzed the sequence similarity of CaBURP genes with matching genes in the selected comparative species. This identified significant regions of similarity on the chromosomes of various species, such as *V. vinifera*, S. lycopersicum, *P. vulgaris*, *A. thaliana*, etc. The observed syntenic association with BURP genes in other plant species suggests functional conservation. It implies that these genes might have important roles in various biological processes that are conserved across different plant species. Such conserved genes may play crucial roles in stress responses or other essential functions. The identification of syntenic regions between CaBURP genes and those in other plant species provides insights into the evolutionary relationships and genetic relatedness among these genes. Understanding such relationships can help in inferring the evolutionary history of BURP genes in different plant lineages. The map of CaBURP genes and their syntenic associations with other species provides valuable genomic resources for further functional studies. Researchers can use this information to explore the roles of BURP genes in various biological processes, including stress responses and development, in chickpea and related plant species. These findings suggest the presence of conserved BURP domain among various plant species adapted to perform multidimensional roles. Conclusively, the mapping of CaBURP genes and their syntenic associations with other plant species sheds light on the distribution and potential functional significance of these genes in *Cicer arietinum*. This information contributes to our understanding of the genetic landscape of BURP genes in chickpea and their evolutionary relationships with other plants. Further studies on these genes can elucidate their specific roles in stress tolerance, growth, and development, potentially leading to practical applications in agriculture and crop improvement.

Eventually, collinearity analysis describes the results of a study that investigated the phylogenetic mechanisms of the BURP family by comparing *C. arietinum* (chickpea) with two model species, *A. thaliana* and *M. truncatula* (barrel medick). We constructed a collinearity diagram to analyze the relationships between BURP genes in these species. The collinearity analysis revealed several conserved genomic regions and potential functional relationships among BURP genes, indicating their significant roles in evolution. The collinearity diagram provides valuable insights into the phylogenetic relationships among BURP genes in *C. arietinum*, *A. thaliana*, and *M. truncatula*. The conserved gene pairs and shared evolutionary history suggest that these genes might have originated from a common ancestor and have been retained in different lineages over time. The collinearity observed between certain BURP genes in *C. arietinum* and the model species (*A. thaliana* and *M. truncatula*) indicates the conservation of genomic regions. These regions likely contain important functional elements that have been preserved across species, implying the biological significance of these genes. The collinearity between BURP genes in *C. arietinum* and the model species suggests potential functional relationships between these genes. Genes that are collinear are likely to share similar functions or be involved in related biological processes, making them interesting candidates for further functional studies. The collinear relationships identified in this study highlight the conservation of specific BURP genes across different species. This conservation implies that these genes have essential roles in the biology of these plants and have been maintained throughout evolution. The use of *A. thaliana* and *M. truncatula* as model species is valuable for this analysis. Both species are well-studied and widely used in plant research. Their well-annotated genomes and extensive genetic resources facilitate the investigation of gene functions and regulatory mechanisms in other plant species, including chickpea. The collinearity between BURP genes from different species indicates potential evolutionary events such as gene duplications, rearrangements, and translocations. These events contribute to the diversity of gene families and the evolution of plant genomes. Understanding the relationships between BURP genes in chickpea and model species may have practical applications in crop improvement. Shared functional elements and conserved regions could be targeted for genetic engineering and breeding programs to enhance stress tolerance, growth, and other desirable traits in crops.

In conclusion, the collinearity analysis of BURP genes in *C. arietinum*, *A. thaliana*, and *M. truncatula* reveals conserved genomic regions and potential functional relationships. This information contributes to our understanding of the evolutionary history and functional significance of BURP genes in different plant species. Further studies on these conserved genes can provide valuable insights into their roles in stress responses, development, and other biological processes, with potential applications in agriculture and biotechnology. The findings for collinearity analysis with *Arabidopsis* indicate the presence of conserved genomic regions and evolutionary associations between the two species. In summary, the collinearity observed between *Arabidopsis* and *C. arietinum* genomes in relation to BURP genes on different chromosomes points towards shared evolutionary history and conserved genomic regions. This collinearity data provides valuable insights into the genetic relationships and potential functional similarities between *Arabidopsis* and *C. arietinum* in the context of BURP genes. Similarly, the collinearity data between *M. truncatula* and *C. arietinum* genomes regarding BURP genes on different chromosomes provides valuable insights into the conservation and evolutionary relationships of these genes across the two plant species. These collinear relationships highlight potential functional significance and shared evolutionary history of BURP genes between *M. truncatula* and *C. arietinum.*

## Conclusion:

In conclusion, this comprehensive study sheds light on the intricate world of BURP domain-containing proteins unique to plants. Through network analysis, we uncovered diverse associations of BURP proteins with other proteins and functional terms, providing valuable insights into their multifaceted roles in plant development and stress responses. Gene expression analysis under salinity stress revealed tissue-specific responses and potential involvement of BURP proteins in stress adaptation. Additionally, synteny and collinearity analyses unveiled conserved genomic regions and evolutionary relationships, highlighting the significance of these genes across plant lineages. These findings deepen our understanding of BURP protein functions and pave the way for practical applications in agriculture and biotechnology.

## Statements and declarations:

The authors have no relevant financial or non-financial interest to disclose.

## Ethical approval:

Authors declare that the study has not used any human and animals in experiments

## Authors' contributions:

K.R.S and S.T conceived the idea and provided critical inputs to the concept. K.R.S. planed the experiment. K.R.S., C. B and S.T generated the data. S.T. executes the computational analysis and wrote the original manuscript. K.R.S., C.B., S.T., M.H., A.K.K., N.C.G., N.K., G.P Dixit contributed to edit the manuscript. All authors contributed to the final reading and approved the submitted version.

## Data Availability Statement:

The data analyzed in this study was obtained from assembled and annotated proteins of *Cicer arietinum* available in Phytozome database (https://phytozome-next.jgi.doe.gov/) and NCBI (https://www.ncbi.nlm.nih.gov). Raw data for analysis was obtained from the Gene Expression Omnibus database (https://www.ncbi.nlm.nih.gov/geo/).

## Consent for publication:

All the authors have consent for publication. All authors have read and agreed to the published version of the manuscript.

## Figures and Tables

**Table 1:** Description of major BURP interactions in *Cicer arietinum*

**Association**	**number of nodes**	**number of edges**	**average node degree**	**avg. local clustering coefficient**	**expected number of edges**	**PPI enrichment p-value**
1	11	18	3.27	0.895	10	0.0157
2	11	32	5.82	0.905	29	0.33
3	11	23	4.18	0.793	11	0.00107
4	11	17	3.09	0.893	10	0.0295
5	11	38	6.19	0.806	12	3.44E-09
6	11	12	2.18	0.883	10	0.313
7	4	3	1.5	0.75	3	0.563

**Table 2:** Major Gene Ontology descriptions and associated KEGG pathway details for various BURP associations

**category**	**#term ID**	**term description**	**observed gene count**	**background gene count**	**false discovery rate**	**matching proteins in your network (IDs)**
GO Component	GO:0005840	Ribosome	5	413	6.30E-04	3827.XP_004490515.1,3827.XP_004498645.1,3827.XP_004500395.1,3827.XP_004502806.1,3827.XP_004512258.1
GO Function	GO:0004349	Glutamate 5-kinase activity	3	6	1.27E-05	3827.XP_004491997.1,3827.XP_004503180.1,3827.XP_004506632.1
GO Function	GO:0004350	glutamate-5-semialdehyde dehydrogenase activity	3	6	1.27E-05	3827.XP_004491997.1,3827.XP_004503180.1,3827.XP_004506632.1
GO Function	GO:0003735	Structural constituent of ribosome	5	368	7.60E-04	3827.XP_004490515.1,3827.XP_004498645.1,3827.XP_004500395.1,3827.XP_004502806.1,3827.XP_004512258.1
GO Function	GO:0010436	Carotenoid dioxygenase activity	2	6	1.30E-03	3827.XP_004512308.1,3827.XP_004512437.1
GO Process	GO:0055129	L-proline biosynthetic process	3	8	6.84E-05	3827.XP_004491997.1,3827.XP_004503180.1,3827.XP_004506632.1
GO Process	GO:0010148	Transpiration	2	8	8.50E-03	3827.XP_004494278.1,3827.XP_004497624.1
GO Process	GO:0050794	Regulation of cellular process	11	5110	1.90E-04	3827.XP_004487248.1,3827.XP_004494007.1,3827.XP_004497360.1,3827.XP_004498687.1,3827.XP_004499691.1,3827.XP_004500901.1,3827.XP_004507416.1,3827.XP_004507701.1,3827.XP_004508282.1,3827.XP_004508315.1,3827.XP_004513027.1
GO Process	GO:0006355	Regulation of transcription, DNA-templated	7	2175	1.59E-02	3827.XP_004487248.1,3827.XP_004494007.1,3827.XP_004497360.1,3827.XP_004499691.1,3827.XP_004500901.1,3827.XP_004507416.1,3827.XP_004508315.1
GO Process	GO:0070829	Heterochromatin maintenance	2	12	1.59E-02	3827.XP_004487248.1,3827.XP_004494007.1
GO Process	GO:0042752	Regulation of circadian rhythm	2	38	4.19E-02	3827.XP_004507701.1,3827.XP_004508282.1
GO Process	GO:0006412	Translation	5	562	1.64E-02	3827.XP_004490515.1,3827.XP_004498645.1,3827.XP_004500395.1,3827.XP_004502806.1,3827.XP_004512258.1
GO Process	GO:0009904	Chloroplast accumulation movement	2	17	3.23E-02	3827.XP_004501570.1,3827.XP_004509626.1
GO Process	GO:0009903	Chloroplast avoidance movement	2	19	3.40E-02	3827.XP_004501570.1,3827.XP_004509626.1
GO Process	GO:0016121	Carotene catabolic process	2	5	2.60E-03	3827.XP_004512308.1,3827.XP_004512437.1
KEGG	cam00330	Arginine and proline metabolism	3	55	2.70E-04	3827.XP_004491997.1,3827.XP_004503180.1,3827.XP_004506632.1
KEGG	cam01230	Biosynthesis of amino acids	3	214	7.10E-03	3827.XP_004491997.1,3827.XP_004503180.1,3827.XP_004506632.1
KEGG	cam03010	Ribosome	5	291	1.41E-05	3827.XP_004490515.1,3827.XP_004498645.1,3827.XP_004500395.1,3827.XP_004502806.1,3827.XP_004512258.1
KEGG	cam00906	Carotenoid biosynthesis	2	36	1.90E-03	3827.XP_004512308.1,3827.XP_004512437.1
